# Supplementary material for: The Arctic Ocean is a net sink for anthropogenic lead deposited into the Atlantic Ocean
Source: Nat Commun. 2025 Dec 17;16:11238. doi: 10.1038/s41467-025-67620-9 (PMC12714867; doi:10.1038/s41467-025-67620-9)
Supplement: Supplementary file 1 — Supplementary Information [file 41467_2025_67620_MOESM1_ESM.pdf]

Supplementary Information

to

The Arctic Ocean is a net sink for anthropogenic lead deposited into the Atlantic Ocean

Stephan Krisch[1,2*], Arianna Olivelli[3,4‡], Loes J.A. Gerringa[5], Rob Middag[5,6], Birgit Rogalla[7,8], Eric P. Achterberg[2]

[1] Technical University of Braunschweig, Braunschweig, Germany. [2] GEOMAR Helmholtz Centre for Ocean Research Kiel, Kiel, Germany. [3] Department of Earth Science & Engineering, Imperial College London, London, United Kingdom. [4] Grantham Institute for Climate Change and the Environment, Imperial College London, London, United Kingdom.[5] NIOZ Royal Netherlands Institute for Sea Research, Den Burg, The Netherlands. [6] Centre for Isotope Research, University of Groningen, Groningen, The Netherlands. [7] Department of Earth, Ocean and Atmospheric Sciences, University of British Columbia, Vancouver, Canada. [8] British Antarctic Survey, Cambridge, United Kingdom. [‡] Now at: Flanders Marine Institute (VLIZ), Ostend, Belgium.

[*]*Correspondence to*: Stephan Krisch (stephan.krisch@tu-braunschweig.de)

Methods

Fram Strait, Barents Sea Opening and Canadian Arctic stations

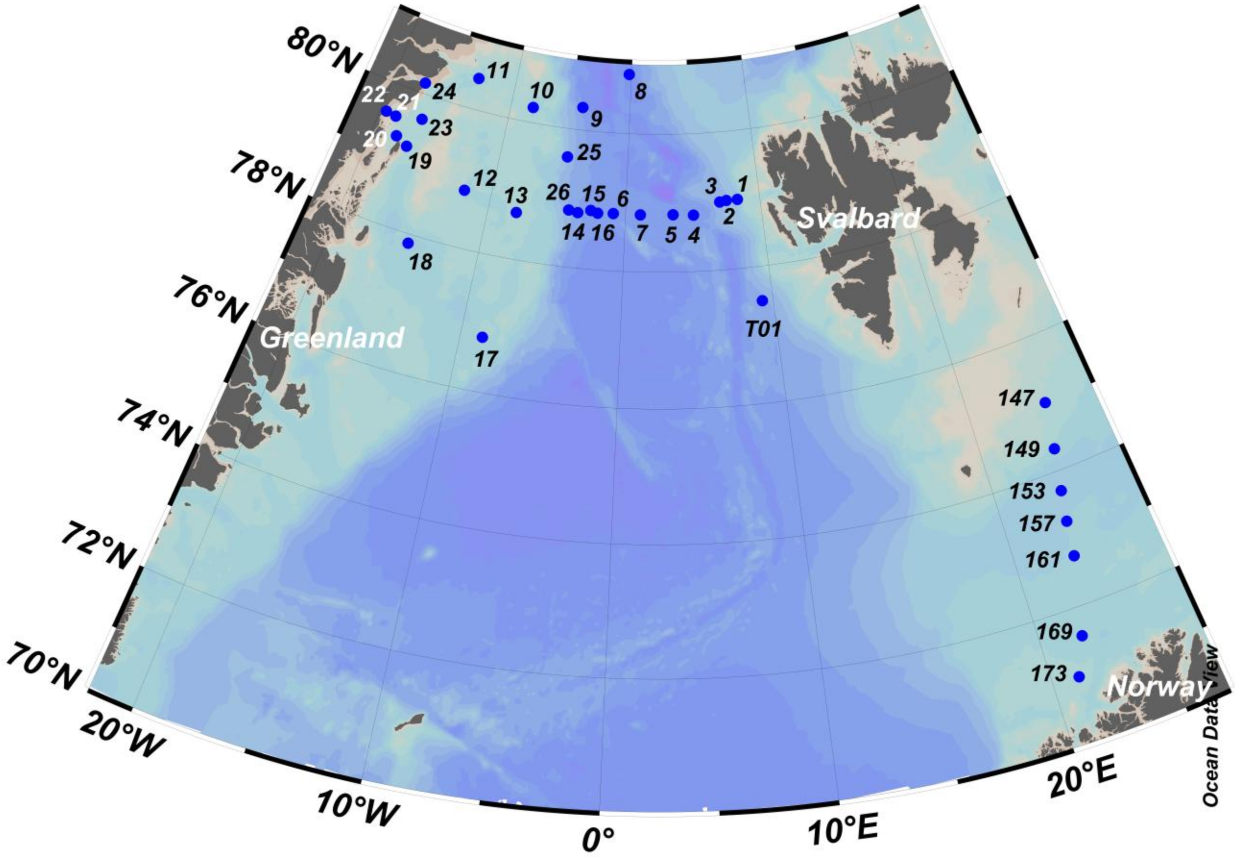

Supplementary Figure 1: Bathymetry map depicting stations sampled for dissolved Pb in (a) the Fram Strait during expedition PS100/GN05 in July-September 2016 (stations 1-26 and test station T01), and (b) the Barents Sea Opening during expedition PS94/GN04 in October 2015 (stations 147-173).

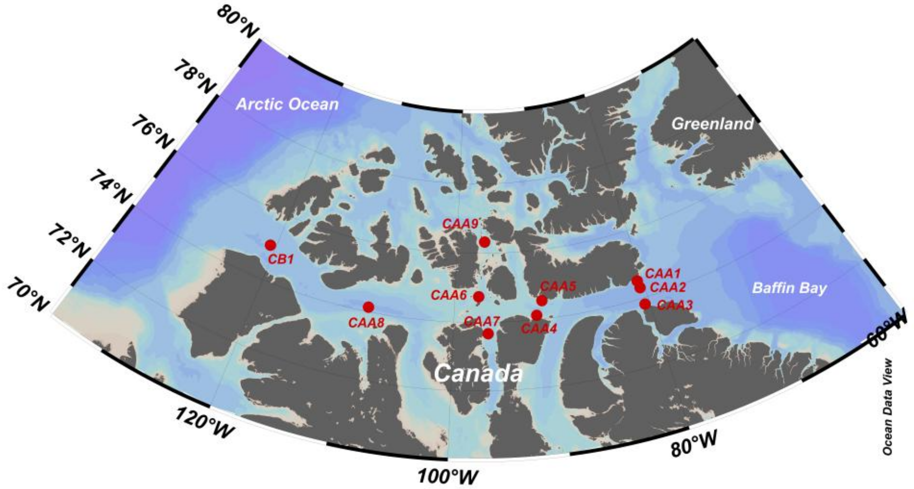

Supplementary Figure 2: Bathymetry map depicting stations sampled for dissolved Pb in the Canadian Arctic Archipelago (CAA) during expedition GN02/GN03 in August-September 2015.

Quality assurance

Validation of method accuracy for dissolved Pb analyses was achieved through the reference material SAFe S and GSC for Fram Strait samples (PS100, GN05) and SAFe D1 and GSP for Barents Sea samples (PS94, GN04) (Supplementary Table 1; ref. [1]). Method sensitivity for dissolved Pb analysis was monitored through SeaFAST-ICP-MS procedural blanks following ref.[2] for PS100/GN05, and procedural blanks plus an internal laboratory standard of North Atlantic Deep Water (NADW) for PS94/GN04 (Supplementary Table 2). Validation of method accuracy for dissolved Pb isotope composition analyses was achieved through repeat measurements of standard reference material NIST SRM 981 (Supplementary Table 3).

Supplementary Table 1: Analyzed reference materials SAFe S and GSC (entire PS100/GN05 dataset), and SAFe D1 and GSP (entire PS94/GN04 dataset) for seawater dPb analysis, n = number of measurements. Standard deviation is reported as 1σ. GSC consensus values taken from ref. [3] but refer to the labile fraction (without UV oxidation).

**Reference Material**	**Consensus value**^{A}	**Reported value**
SAFe S (#273)	49.2 ± 2.3 pmol·L^{-1}	48.9 ± 2.9 pmol·L^{-1} (n = 11)
GSC (#21, #159)	40 ± 2 pmol·L^{-1}	37 ± 2 pmol·L^{-1} (n = 7)
SAFeD1 (#599)	28.4 ± 2.7 pmol·L^{-1}	25 ± 1 pmol·L^{-1} (n = 6)
GSP (#227)	66.4 ± 1.2 pmol·L^{-1}	62 ± 1 pmol·L^{-1} (n=3)

^{A} includes conversion from pmol·kg^{-1} using density of 1.026 kg·L^{-1}

Supplementary Table 2: Method sensitivity for seawater Pb analyses determined as procedural blanks for the PS100/GN05 and PS94/GN04 dataset, n = number of measurements. Standard deviation is reported as 1σ.

SeaFAST-ICP-MS System blank (GN05)	0.7 ± 0.3 pmol·L^{-1} (n = 555)
SeaFAST-ICP-MS System blank (GN04)	1.3 ± 1 pmol·L^{-1} (n = 16)

Supplementary Table 3: Analyzed reference material NIST SRM 981 for measurements of dPb isotope composition for pooled seawater samples from GEOTRACES expedition GN05/PS100. Consensus values taken from refs. [4] (A) and [5] (B). Standard deviations are reported as 2σ. n.d. = not determined.

	**^{206}Pb/^{204}Pb**	**^{207}Pb/^{204}Pb**	**^{208}Pb/^{204}Pb**	**^{206}Pb/^{207}Pb**	**^{208}Pb/^{207}Pb**
**Consensus value A**	16.941 ± 0.002	15.496 ± 0.002	36.722 ± 0.004	n.d.	n.d.
**Consensus value B**	16.935 ± 0.009	15.497 ± 0.008	36.723 ± 0.019	1.0931 ± 0.0001	2.3697 ± 0.0005
**Reported value (n=10)**	16.939 ± 0.011	15.498 ± 0.010	36.723 ± 0.033	1.09295 ± 0.00012	2.36953 ± 0.00014

Dissolved Pb distribution in the Fram Strait

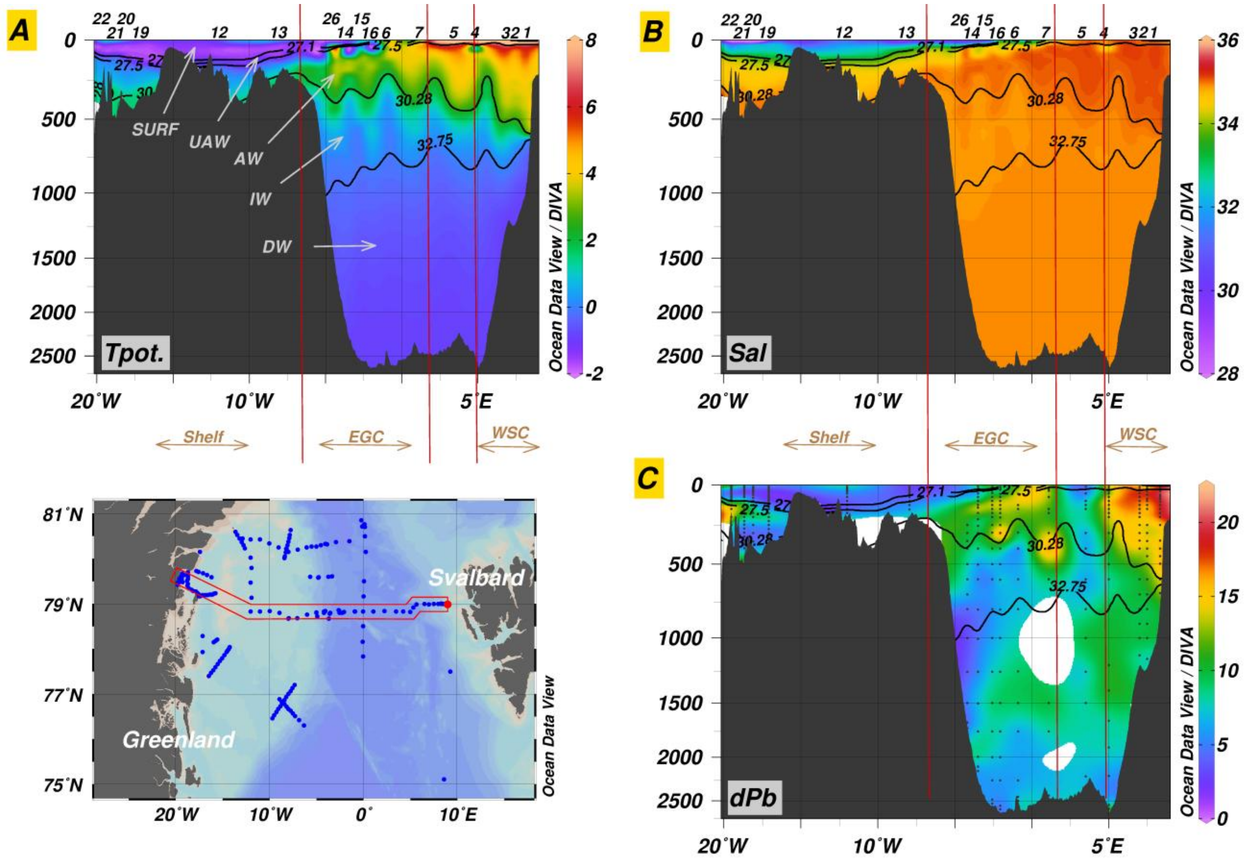

Supplementary Figure 3: Distributions of (A) potential temperature ($T_{pot.}$, in °C), (B) salinity (Sal) and (C) dissolved Pb (dPb, in pmol·L^{-1}) in Fram Strait at 79°N. Section plotted as indicated in bottom left map. Station numbers indicated in top panel. Isopycnal surfaces (black contours), derived from CTD measurements, distinguish between water masses: Surface Water (SURF, $\sigma_0 < 27.1$ kg·m^{-3}), Upper Atlantic Water (UAW, 27.10σ_0–27.50σ_0 kg·m^{-3}), Atlantic Water (AW, 27.50σ_0–30.28$\sigma_{0.5}$ kg·m^{-3}), Intermediate Water (IW, 30.28$\sigma_{0.5}$– 32.75$\sigma_{1.0}$ kg·m^{-3}) and Deep Water (DW) (DW, $\sigma_{1.0} >$ 32.75 kg·m^{-3}). Vertical red lines delineate the West Spitsbergen Current (WSC, >5°E), the Middle (2–5°E), the East Greenland Current (EGC, 6.5°W–2°E) and the NE Greenland Shelf (>6.5°W) following the definitions of ref. [6].

Dissolved Pb distribution in the Barents Sea Opening

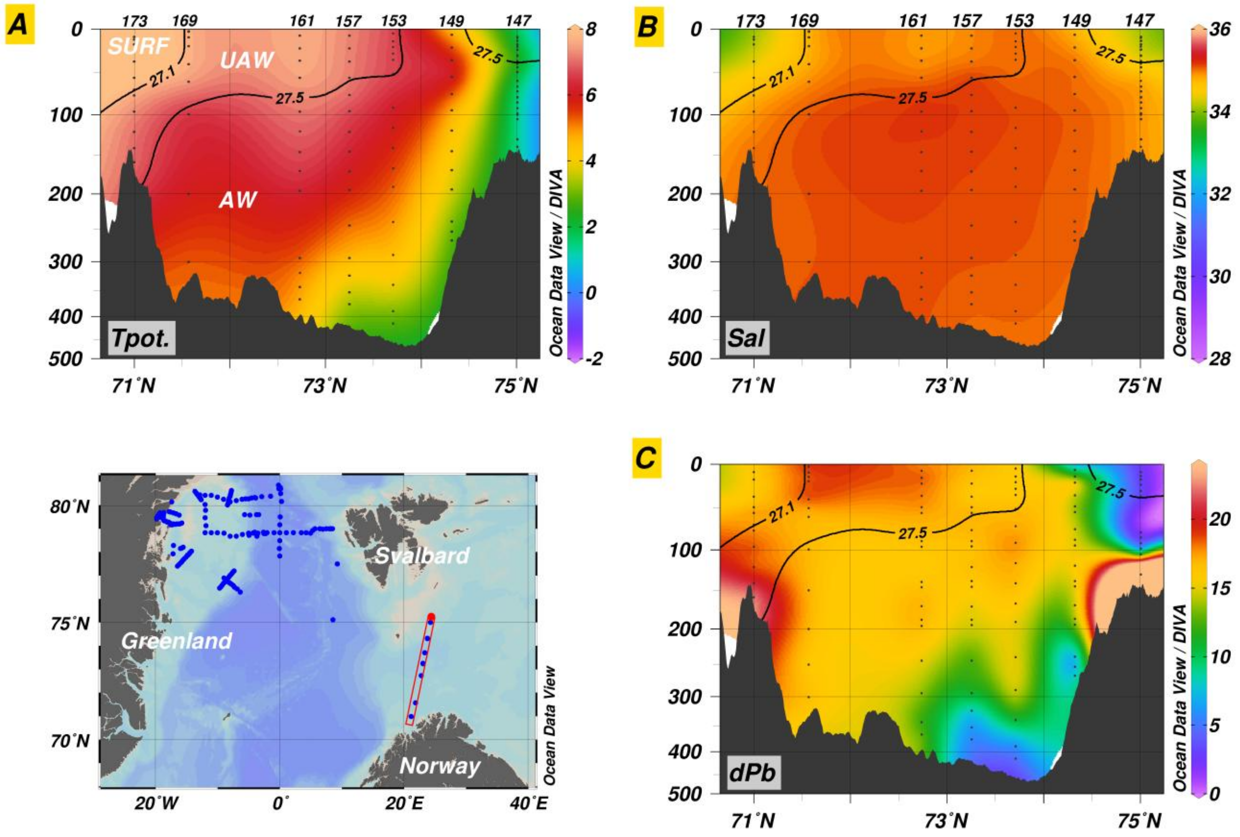

Supplementary Figure 4: Distributions of (A) potential temperature ($T_{pot.}$, in °C), (B) salinity (Sal) and (C) dissolved Pb (dPb, in pmol·L^{-1}) in the Barents Sea Opening. Section indicated in bottom left map. Station numbers indicated in top panel. Isopycnal surfaces (black contours), derived from CTD measurements, distinguish between water masses: Surface Water (SURF, $\sigma_0 < 27.1$ kg·m^{-3}), Upper Atlantic Water (UAW, 27.10σ_0–27.50σ_0 kg·m^{-3}) and Atlantic Water (AW, 27.50σ_0–30.28$\sigma_{0.5}$ kg·m^{-3}) following the definitions of ref. [6].

Supplementary Table 4: Compilation of dissolved Pb (dPb) data for Atlantic Water (AW) and Arctic Atlantic Water (AAW) in the Central Arctic Ocean obtained during GEOTRACES expeditions GN02/03 (Canada Basin, ref.[7]), GN04 (Central Arctic Ocean and Barents Sea Opening, ref.[8]) and GN05 (Fram Strait, ref.[9]). Stations numbers refer to original data publications. Dissolved Pb concentration averages (AVG) and standard deviations (STD, 1σ) are in pmol·L^{-1}; n = number of datapoints. GN02/03 dPb data was converted from pmol·kg^{-1} to pmol·L^{-1} by the density of seawater (1.023 kg·L^{-1} as per ref.[10]). Depth indicates the depth range (Min-Max) where AW and AAW is present and dPb data above limit of detection (LOD) is available. Comment section indicates the number of measurements that were <LOD.

Domain		Expedition	Station	Lat [°N]	Lon [°E]	Depth [m]		dPb			
						Min	Max	AVG	STD	n	Comments
Fram Strait WSC	AW	GN05	1-4	79	5.0-8.3	59	600	15.4	2.3	41	
Barents Sea Opening	AW	GN04	149-173	71.0-74.3	21.2-23.8	3	445	15.5	3.3	102	
Nansen Basin	AAW	GN04	32	81.9	30.9	153	731	15.0	7.1	7	
Nansen Basin	AAW	GN04	50	84.4	30.5	95	733	9.6	0.8	8	
Nansen Basin	AAW	GN04	54	85.1	42.4	144	733	7.8	0.7	7	
Nansen Basin	AAW	GN04	58	85.3	59.8	119	734	6.5	0.9	8	
Nansen Basin	AAW	GN04	64	86.4	60.2	118	684	6.1	2.5	14	
Amundsen Basin	AAW	GN04	69	87.0	58.7	144	734	3.9	1.0	3	2/5<LOD
Amundsen Basin	AAW	GN04	70	87.0	55.4	141	734	4.7	1.4	4	
Amundsen Basin	AAW	GN04	87	89.9	-120.2	293	734	2.7		1	2/3 <LOD
Makarov Basin	AAW	GN04	91	89.2	-116.7	191	783	3.1	0.3	2	8/10 <LOD
Makarov Basin	AAW	GN04	96	88.4	-124.9	189	733	3.1	0.0	2	2/4 <LOD
Canada Basin	AAW	GN02/03	CB4	75.0	-150.0	500	1000	4.5	0.8	4	
Fram Strait	AAW	GN05	9	80.3	-4.0	200	601	2.7	0.5	5	
Fram Strait	AAW	GN05	14	78.8	-3.5	303	602	6.7	1.9	4	
Fram Strait	AAW	GN05	16	78.8	-2.0	401	802	7.7	1.3	4	
Fram Strait	AAW	GN05	25	79.6	-4.8	175	801	3.6	0.7	9	

Supplementary Table 5: Compilation of mode ages (t_{mode}) for Atlantic Water (AW) and Arctic Atlantic Water (AAW) in the Central Arctic Ocean from ref.[11]. The t_{mode} indicates the most probable age for circulation of AAW through the Central Arctic Ocean [12]. (*) Atlantic Water entering the Arctic Ocean through Fram Strait (as part of the West Spitsbergen Current, WSC) and the Barents Sea Opening is defined as t_{mode} = 0. Only t_{mode} ages considered herein where dPb data is available (Supplementary Table 4). No t_{mode} data for the Makarov Basin available. Mode age averages (AVG) and standard deviations (STD, 1σ) are in years; n = number of data points.

Domain		Expedition	Station	Lat [°N]	Lon [°E]	Depth [m]	t_{mode}		
							AVG	STD	n
Fram Strait WSC	AW	GN05					0*		
Barents Sea Opening	AW	GN04					0*		
Nansen Basin	AAW	GN04	50	84.4	30.4	300+501	8.1	2.4	2
Nansen Basin	AAW	GN04	58	85.3	59.9	300+500	5.9	1.4	2
Amundsen Basin	AAW	GN04	68	87.0	58.8	300+501	8.8	0.0	2
Canada Basin	AAW	GN01	56	75.1	-150.4	503+800	27.6	1.0	2
Fram Strait	AAW	GN05	9	80.3	-4.0	301+470+800	17.8	1.5	3
Fram Strait	AAW	GN05	14	78.8	-3.6	301+500+802	14.5	1.3	3
Fram Strait	AAW	GN05	16	78.8	-2.2	301+500+802	10.1	3.8	3
Fram Strait	AAW	GN05	25	79.6	-4.8	301+501+800	17.6	2.6	3

Atlantic Water dPb concentration vs transit time (tracer age)

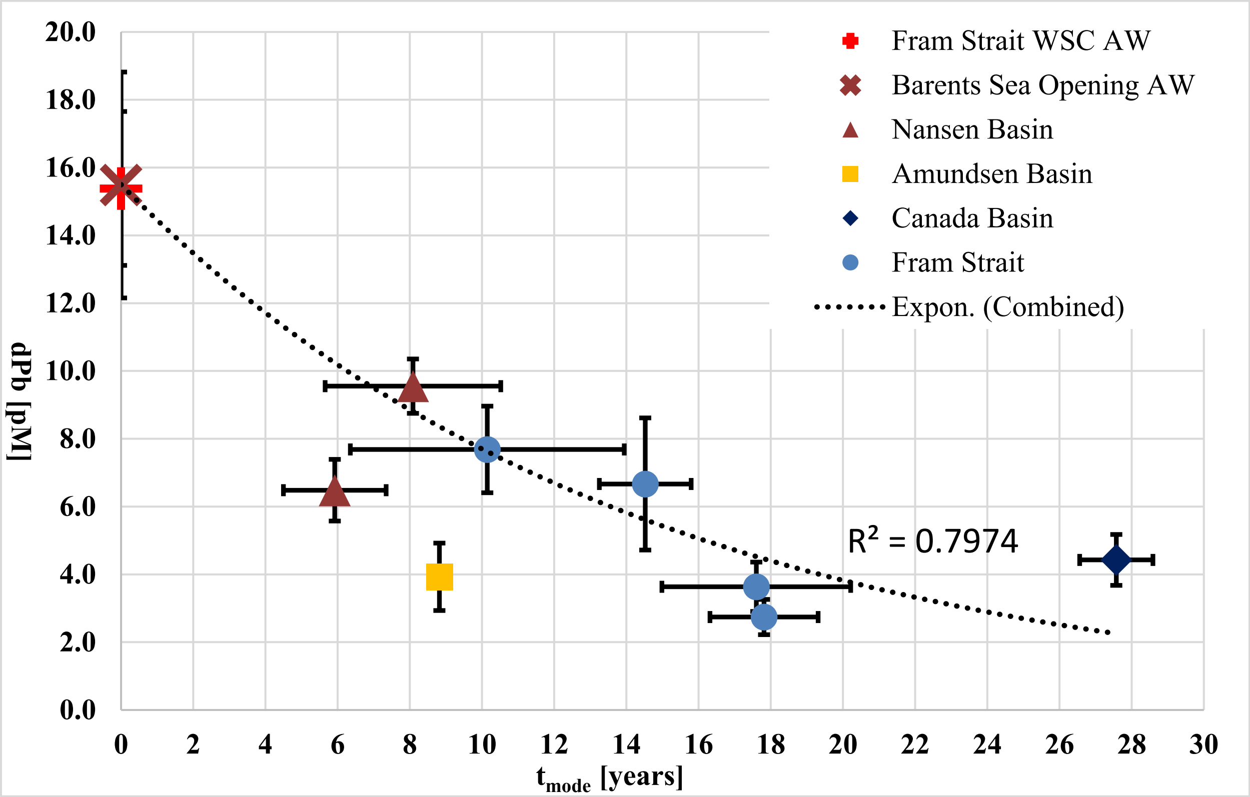

Supplementary Figure 5: Dissolved Pb concentrations in Atlantic and Arctic Atlantic Water of the study region. Correlation of dissolved Pb concentrations (dPb, in pmol·L^{-1}, pM) *versus* mode age of Arctic Atlantic Water (t_{mode}) in the Nansen Basin (brown triangle), Amundsen Basin (yellow square), Canada Basin (blue diamond) and Fram Strait (blue dots). Dissolved Pb concentrations from GEOTRACES expeditions GN02/03 (ref. [7]), GN04 (ref. [8]) and GN05 (ref. [9]) are given in Supplementary Table 4; t_{mode} derived from ^{129}I and ^{236}U measurements as per ref.[11] are summarized in Supplementary Table 5. Atlantic Water entering the Arctic Ocean through Fram Strait (red cross) and the Barents Sea Opening (brown cross) functions as reference and is defined as $t_{mode} = 0$. Whiskers show the standard deviations to the mean dPb concentration and the mean mode age. Quasi-exponentially decreasing dPb concentrations (black dotted line) with increasing age of Arctic Atlantic Water to the function of $y = 15.5 \cdot e^{-0.07x}$ (0.80 R^2) are observed.

Dissolved Pb isotope composition

Supplementary Table 6: Compilation of dissolved Pb (dPb) isotope composition data from GEOTRACES expeditions GN01 (ref. [13]), GN02/03 (ref. [14]) and GN05 (ref. [15]). GN05 samples were pooled according to water mass properties of Atlantic Water (AW), Recirculating Atlantic Water (RAW) and Arctic Atlantic Water (AAW) after Rudels et al. (2005)[16] and were measured in this study. Atlantic Water entering the Arctic Ocean through Fram Strait ('Fram Strait Branch', FSB, defined as per ref. [17]) is traced along its flow path across the Amundsen Basin (AB), Makarov Basin (MB), and Canada Basin (CB). Station numbers are given as in original data publications. Average concentrations of dPb are given in pmol·L^{-1} (pM). GN01 and GN02/03 dPb concentration data was converted from pmol·kg^{-1} to pmol·L^{-1} by the density of seawater (1.023 kg·L^{-1} as per ref.[10])

Cruise	Station		Water Mass	Lat [°N]	Lon [°E]	Depth [m]	dPb [pM]	^{206}Pb/^{204}Pb	^{206}Pb/^{207}Pb	^{207}Pb/^{204}Pb	^{208}Pb/^{204}Pb	^{208}Pb/^{206}Pb	^{208}Pb/^{207}Pb
GN05	T01	FS	AW	77.497	9.277	**39-373**	**17.7**	18.330	1.177	15.567	38.087		2.447
GN05	1	FS	AW	79.001	8.328	**71-449**	**14.6**	18.404	1.179	15.615	38.240		2.449
GN05	2	FS		79.000	7.498	**60-500**							
GN05	3	FS	AW	78.986	6.994	**74-400**	**14.5**	18.415	1.179	15.623	38.268		2.449
GN05	4	FS		78.819	4.997	**75-401**							
GN05	14	FS	RAW	78.804	-3.510	**125-255**	**11.6**	18.420	1.179	15.624	38.283		2.450
GN05	15	FS		78.854	-2.567	**75-300**							
GN01	32	AB	FSB	89.991	35.646	**349**	**4.4**	18.610	1.187				2.451
GN01	32	AB	FSB	89.991	35.646	**424**	**3.9**	18.600	1.186				2.452
GN01	32	AB	FSB	89.991	35.646	**499**	**5.3**	18.730	1.193				2.453
GN01	32	AB	FSB	89.991	35.646	**499**	**3.6**	18.610	1.185				2.451
GN01	32	AB	FSB	89.991	35.646	**749**	**2.0**	18.470	1.181				2.451
GN01	30	MB	FSB	87.586	179.793	**374**	**1.4**	18.430	1.194				2.459
GN01	30	MB	FSB	87.586	179.793	**499**	**3.4**	18.690	1.194				2.452
GN01	30	MB	FSB	87.586	179.793	**499**	**2.3**	18.280	1.185				2.454
GN01	30	MB	FSB	87.586	179.793	**799**	**2.5**	18.400	1.188				2.455
GN01	43	MB	FSB	85.145	210.004	**599**	**3.9**	18.770	1.195				2.453
GN01	43	MB	FSB	85.145	210.004	**699**	**3.6**	18.500	1.186				2.448
GN01	19	CB	FSB	79.986	184.985	**449**	**0.9**	18.700	1.214				2.470
GN01	19	CB	FSB	79.986	184.985	**499**	**1.5**	18.550	1.196				2.456
GN01	19	CB	FSB	79.986	184.985	**499**	**1.1**	18.530	1.195				2.457
GN01	19	CB	FSB	79.986	184.985	**584**	**1.9**	18.820	1.212				2.466
GN01	19	CB	FSB	79.986	184.985	**699**	**1.7**	18.780	1.212				2.468

GN01	19	CB	FSB	79.986	184.985	**849**	**1.5**	18.610	1.197				2.459
GN01	48	CB	FSB	80.425	210.628	**495**	**4.1**	18.790	1.196				2.452
GN01	48	CB	FSB	80.425	210.628	**710**	**2.3**	18.600	1.190				2.450
GN01	52	CB	FSB	77.484	212.052	**901**	**2.1**	18.640	1.189				2.452
GN01	57	CB	FSB	73.414	203.256	**661**		18.570	1.194				2.455
GN01	57	CB	FSB	73.414	203.256	**751**	**1.1**	18.550	1.199				2.454
GN01	57	CB	FSB	73.414	203.256	**951**		18.580	1.193				2.449
GN03	CB2	CB	FSB	75.806	230.765	**800**	**5.8**		1.139			2.120	
GN03	CB3	CB	FSB	76.993	219.946	**800**	**4.4**		1.153			2.104	
GN03	CB3	CB	FSB	76.993	219.946	**1000**	**3.3**		1.153			2.104	
GN03	CB4	CB	FSB	75.001	209.999	**800**	**5.3**		1.146			2.111	
GN03	CB4	CB	FSB	75.001	209.999	**1000**	**3.3**		1.168			2.084	
GN05	8	FS		80.853	-0.206	**175-501**							
GN05	9	FS		80.326	-4.009	**200-600**							
GN05	14	FS	AAW	78.804	-3.510	**303-601**	**4.6**	18.378	1.178	15.606	38.201		2.448
GN05	25	FS		79.589	-4.772	**175-801**							
GN05	26	FS		78.829	-4.188	**501-700**							

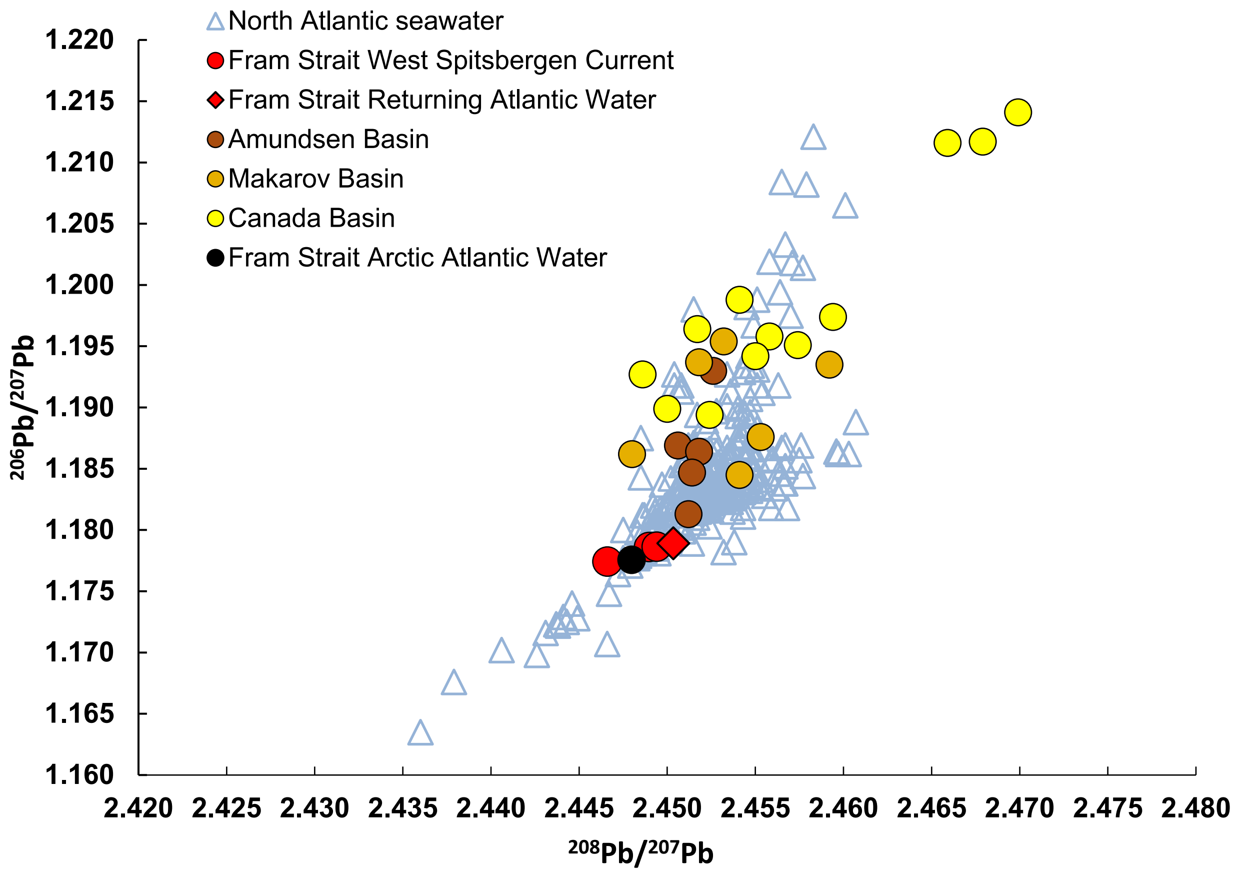

Supplementary Figure 6: Three-isotope plot showing dissolved Pb (dPb) isotopic composition ($^{206}Pb/^{207}Pb$ vs $^{208}Pb/^{207}Pb$) for Atlantic Water samples collected in the Arctic Ocean (filled dots), Returning Atlantic Water (also termed 'Recirculating Atlantic Water') in Fram Strait (red diamond) and seawater samples from the North Atlantic (blue open triangles). Only Fram Strait Branch Atlantic Water is considered here (as compiled in Supplementary Table 6). North Atlantic seawater data obtained from ref. [18].

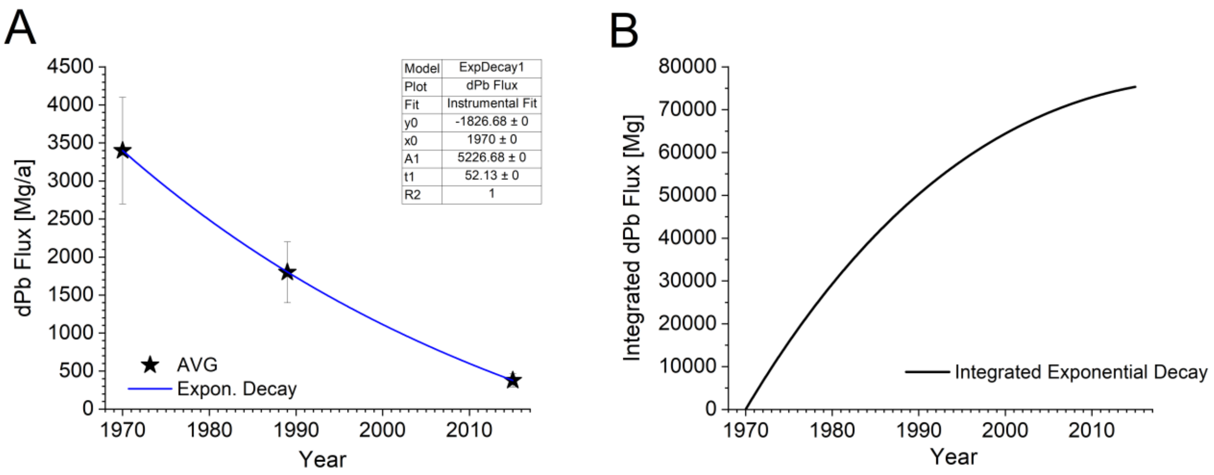

Supplementary Figure 7: Transport estimates of dissolved Pb (dPb) from the Atlantic into the Arctic Ocean across the gateways of Fram Strait, the Barents Sea Opening, and the Canadian Arctic Archipelago between 1970 and 2015/2016. (A) Estimates of Arctic-Atlantic dPb fluxes (black stars, in $Mg \cdot a^{-1}$) derived from ref. [19] for 1970, ref. [18] for 1989, and this study for 2015/2016. Whiskers show the standard deviations to the calculated fluxes. We refer the reader to the main text for information regarding the calculation of estimates. The exponential decay function $y = y_0 + A_1 \cdot e^{-(x-x0)/t1}$ (blue line) and its statistic are presented. (B) Estimate of the integrated net dPb transport across the Arctic-Atlantic gateways from 1970. The integrated net dPb flux is calculated from the exponential decay function presented in (A).

Supplementary Notes

Fram Strait and Barents Sea Opening dPb flux calculations

The dPb flux calculations for the period September 2015-August 2016 are conducted following previously established methods for Hg[20] and dissolved micronutrients including Fe[21] from the same expeditions utilising monthly average volume fluxes in individual water mass layers (defined by isopycnal surfaces) from the period September 2005 to August 2006 (Supplementary Table 7)[6], and average dPb concentrations in water masses of Fram Strait (21 July – 1 September 2016, GEOTRACES cruise GN05) and the Barents Sea Opening (6-9 Oct 2015, GEOTRACES cruise GN04) (Supplementary Table 8). Dissolved Pb data of Fram Strait (stations 1-26, Supplementary Figure 1) is available on Pangaea[9]. Dissolved Pb data from the Barents Sea Opening (Station 147-173, Supplementary Figure 2) has been published by the NIOZ Dataverse[8]. Barents Sea Opening station 147 is excluded from flux calculations owing to strong local influence from Arctic Ocean outflow and sediment resuspension, both of which are not representative of Barents Sea Branch Atlantic Water advection into the Arctic Ocean[22].

Water masses of Fram Strait are the West Spitsbergen Current (>5°E), the Middle section (2-5°E), the East Greenland Current (2°E-6.5°W), and the NE Greenland Shelf (>6.5°W) and are differentiated into layers of Surface Water ($\sigma_0 < 27.1$ kg/m^3), Upper Atlantic Water (27.10σ_0 - 27.50σ_0 kg/m^3), Atlantic Water (27.50σ_0 - 30.28$\sigma_{0.5}$ kg/m^3), Intermediate Water (30.28$\sigma_{0.5}$ - 32.75$\sigma_{1.0}$ kg/m^3), and Deep Water ($\sigma_{1.0} > 32.75$ kg/m^3) by definition of isopycnal surfaces and potential density relative to 0 dbar (σ_0), 500 dbar ($\sigma_{0.5}$) and 1000 dbar ($\sigma_{1.0}$) using GN04 and GN05 CTD-data following ref.[6] (Supplementary Figure 3). The water column of the Barents Sea Opening is analogously distinguished into layers, but treated as one water mass ('Barents Sea Branch') in accordance with ref.[20] (Supplementary Figure 4). Dissolved Pb fluxes are calculated using year-long mean volume transport flows, and mean dPb concentrations for each individual water mass within every layer ('component'). Volume transport flows for each component and each month between September 2005 and August 2006 are summarized in Supplementary Table 7. Mean dPb concentrations of each component in Fram Strait (PS100/GN05) and the Barents Sea Opening (PS94/GN04) are summarized in Supplementary Table 8.

Uncertainties in dPb transport rate for each component are derived from error propagation and include the standard deviation of volume transport (i.e. seasonal variation) and standard deviation of dPb concentrations from section cruises (i.e. without seasonal variation). To establish annual dPb fluxes, we average over monthly mean net dPb flux (Sep-Aug) and report uncertainty as one standard deviation (1σ) of monthly variations following ref.[20]. Supplementary Table 9 summarizes the calculated annual dPb flux through Fram Strait and the Barents Sea Opening.

Supplementary Table 7: Volume transport rates (in Sv = 10^6 $m^3 \cdot s^{-1}$) for the individual components of Surface Water (SURF), Upper Atlantic Water (UAW), Atlantic Water (AW), Intermediate Water (IW) and Deep Water (DW) in Fram Strait and the Barents Sea Opening, extracted from ref.[6]. Negative (positive) values indicate Arctic export to (import from) the high latitude North Atlantic Ocean.

Component		Sep 2005	Oct 2005	Nov 2005	Dec 2005	Jan 2006	Feb 2006	Mar 2006	Apr 2006	May 2006	Jun 2006	Jul 2006	Aug 2006
West Spitsbergen Current	SURF	Absent											
	UAW	Absent											
	AW	3.16	4.09	4.11	3.89	4.56	3.53	3.67	3.94	3.77	2.94	3.49	3.37
	IW	0.99	1.13	0.95	1.58	1.36	1.25	2.39	1.83	1.58	1.42	1.22	0.95
	DW	1.95	2.21	1.91	2.55	2.67	1.98	2.01	3.90	1.77	2.53	1.61	2.32
	**Net**	**6.10**	**7.43**	**6.96**	**8.03**	**8.59**	**6.77**	**8.07**	**9.67**	**7.12**	**6.88**	**6.32**	**6.86**
Middle	SURF	0.00	0.00	0.00	0.00	-0.07	0.00	0.00	0.00	0.00	-0.04	-0.06	-0.08
	UAW	-0.02	-0.11	-0.08	-0.04	0.00	0.00	0.00	-0.02	-0.01	-0.04	-0.01	-0.04
	AW	-1.02	-0.74	0.21	-0.24	0.06	0.71	-0.67	-2.14	0.54	-0.07	0.00	-0.83
	IW	-0.27	0.04	-0.09	-0.15	-0.41	0.32	-1.60	-1.23	-0.32	-0.42	-0.53	-0.36
	DW	-0.93	-0.82	-0.65	-0.85	-2.52	-0.13	-2.18	-2.46	-0.35	-0.49	-0.73	-1.45
	**Net**	**-2.24**	**-1.64**	**-0.61**	**-1.28**	**-2.94**	**0.90**	**-4.45**	**-5.84**	**-0.14**	**-1.06**	**-1.34**	**-2.64**
East Greenland Current	SURF	-0.56	-0.77	-0.98	-0.79	-0.52	-0.68	-0.29	-0.28	-0.56	-0.67	-0.88	-0.77
	UAW	-0.54	-0.43	-0.49	-0.69	-0.81	-0.74	-0.71	-0.60	-0.61	-0.70	-0.39	-0.70
	AW	-0.72	-1.15	-0.99	-1.06	-1.07	-1.18	-1.30	-0.76	-0.49	-0.71	-0.63	-0.84
	IW	-2.90	-2.01	-2.23	-2.50	-2.47	-3.19	-2.30	-1.35	-2.12	-2.79	-2.44	-2.51
	DW	-2.09	-2.41	-2.63	-2.13	-2.17	-1.69	-0.09	-0.87	-1.76	-1.54	-1.59	-0.44
	**Net**	**-6.82**	**-6.77**	**-7.32**	**-7.17**	**-7.05**	**-7.48**	**-4.69**	**-3.85**	**-5.53**	**-6.41**	**-5.93**	**-5.26**
NE Greenland Shelf	SURF	-0.16	-0.07	-0.19	-0.22	-0.86	-1.01	-0.91	-0.19	-0.25	-0.39	-0.53	0.29
	UAW	-0.55	-0.17	0.04	0.05	-0.28	-0.02	0.01	-0.53	-0.19	0.07	0.07	0.26
	AW	0.14	0.19	0.22	0.20	0.04	0.06	0.12	0.10	0.10	0.15	0.11	0.19
	IW	-0.03	-0.01	0.00	0.00	-0.02	-0.01	-0.02	0.00	-0.01	-0.01	0.00	-0.01
	DW	Absent											
	**Net**	**-0.60**	**-0.06**	**0.07**	**0.02**	**-1.10**	**-0.98**	**-0.80**	**-0.61**	**-0.35**	**-0.17**	**-0.35**	**0.73**
Barents Sea Opening	SURF	1.07	0.59	0.52	0.57	0.58	0.49	0.28	0.22	0.16	0.22	0.43	0.81
	UAW	0.81	1.00	1.30	0.96	0.76	0.38	0.23	0.29	0.24	0.58	0.64	0.68
	AW	2.13	0.69	1.35	0.48	2.72	0.97	0.85	0.83	-0.63	2.02	1.56	0.85
	IW	-0.04	-0.27	0.12	0.06	0.21	-0.03	0.08	0.04	0.00	-0.01	-0.10	-0.19
	DW	0.00	0.00	0.00	0.00	0.06	0.03	0.12	0.12	-0.10	-0.06	-0.02	0.00
	**Net**	**3.97**	**2.01**	**3.29**	**2.07**	**4.32**	**1.84**	**1.56**	**1.52**	**-0.33**	**2.74**	**2.51**	**2.14**

Supplementary Table 8: Average dPb concentrations (in pmol·L^{-1} (pM)) in Surface Water (SURF), Upper Atlantic Water (UAW), Atlantic Water (AW), Intermediate Water (IW) and Deep Water (DW) of the (a) West Spitsbergen Current, (b) the 'Middle' section, (c) the East Greenland Current, (d) the NE Greenland Shelf, and (e) the Barents Sea Opening. Water mass definition as per ref.[6]. Mean value ± standard deviation. n = number of datapoints.

**Component**		**dPb [pM]**	**n**
West Spitsbergen Current	SURF	*absent*	
	UAW	17.1 ± 2.1[A]	4
	AW	15.7 ± 2.5 (15.8 ± 2.5)[A]	51 (55)
	IW	9.8 ± 0.9	8
	DW	9.3 ± 2.2	16
Middle	SURF	*absent*	
	UAW	*absent*	
	AW	6.3 ± 1.5	7
	IW	6.8 ± 3.1[B]	43
	DW	11.0 ± 6.8	6
East Greenland Current	SURF	5.7 ± 4.0	13
	UAW	5.5 ± 2.4	8
	AW	9.1 ± 4.1	61
	IW	6.1 ± 3.0	35
	DW	5.9 ± 1.9	53
NE Greenland Shelf	SURF	5.3 ± 4.0	102
	UAW	7.5 ± 3.2	22
	AW	7.0 ± 2.7	54
	IW	5.8 ± 2.7	13
	DW	*absent*	
Barents Sea Opening	SURF	16.8 ± 0.8	6
	UAW	17.8 ± 1.6	32
	AW	14.1 ± 3.4	63
	IW	*absent*	
	DW	*absent*	

[A] Upper Atlantic Water not observed during Sep 2005 – Aug 2006. Upper Atlantic Water dPb concentrations included in AW average and standard deviation (in brackets). [B] Intermediate Water not sampled during GN04. Therefore, mean of West Spitsbergen Current Intermediate Water and East Greenland Current Intermediate Water concentrations.

Supplementary Table 9: Monthly mean estimates of dPb fluxes (in Mg·a^{-1}) through Fram Strait (FS), and the Barents Sea Opening (BSO), including the Fram Strait West Spitsbergen Current (WSC), East Greenland Current (EGC), Middle section (M) and NE Greenland Shelf (NEGS). Fluxes calculated from monthly mean volume transport [6] and mean GN04/05 dPb concentrations in the corresponding components (Supplementary Tables 7 and 8). Contribution of West Spitsbergen Current Upper Atlantic Water, absent during the 2005-2006 observation of volume fluxes, was estimated from assuming proportionality between layer thickness and volume transport from the West Spitsbergen Current Atlantic Water layer. Annual mean net dPb fluxes (AVG) are calculated as average of monthly fluxes; the corresponding uncertainty (STD) is reported as standard deviation (1σ) to monthly variations. Negative (positive) values indicate Arctic export to (import from) the high latitude North Atlantic Ocean.

Month	WSC	EGC	M	NEGS	FS	BSO	FS+BSO
**Sep 15**	508	-280	-121	-27	81	409	489
**Oct 15**	629	-286	-92	-3	247	245	493
**Nov 15**	601	-304	-46	5	257	334	590
**Dec 15**	658	-299	-79	4	283	224	507
**Jan 16**	720	-295	-200	-42	183	428	611
**Feb 16**	565	-315	34	-33	251	188	438
**Mar 16**	654	-209	-256	-26	163	154	317
**Apr 16**	761	-164	-320	-28	249	151	400
**May 16**	598	-224	-18	-14	342	-22	320
**Jun 16**	548	-264	-60	-3	220	271	491
**Jul 16**	536	-243	-80	-10	203	255	458
**Aug 16**	550	-221	-159	31	200	229	429
**AVG**	611	-259	-116	-12	223	239	462
**STD**	74	44	97	20	63	116	86

Canadian Arctic Archipelago dPb fluxes

Transport rates of dissolved Pb (dPb) across the Canadian Arctic were calculated from (a) modeled mean volume fluxes across the Baffin Bay gateways in Lancaster Strait, Nares Strait and Jones Sound, and (b) dPb data at the entrance of Lancaster Strait into Baffin Bay (stations CAA1-CAA3 as per Colombo et al. 2019[7]). Our approach follows calculation of dissolved Fe transport across the Canadian Arctic Archipelago as per ref. [23].

Volume fluxes data

The main flow pathways from the Arctic Ocean through the Canadian Arctic Archipelago (CAA) to Baffin Bay are Lancaster Sound and Nares Strait, and to a lesser extent Jones Sound. We calculated volume transport across sections in these pathways (Supplementary Figure 8) using 5-day average dynamics fields from the reference experiment of the ANHA12 configuration within the Nucleus for European Modeling of the Ocean (NEMO, https://canadian-nemo-ocean-modelling-forum-commuity-of-practice.readthedocs.io/en/latest/Institutions/UofA/Configurations/ANHA12/index.html) between September 2015 and August 2016. Volume fluxes were separated into the upper (depth <47 m), middle (47-92 m), and lower water column (>92 m). Our calculation of volume fluxes falls within the ranges of refs. [24] and [25].

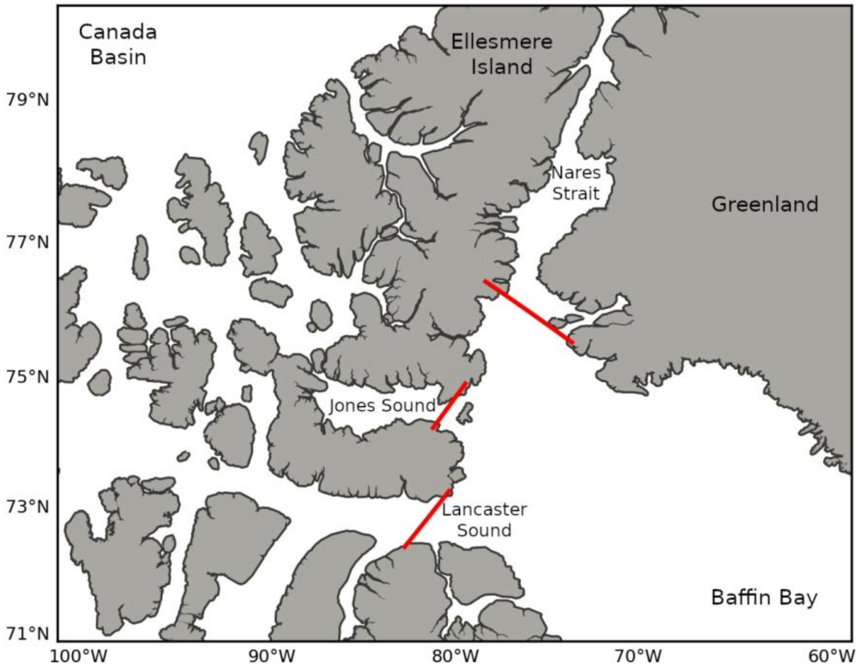

Supplementary Figure 8: Cross-section locations for volume transport calculations in the CAA (red lines). Cross-sections were chosen to run along the model grid for accuracy.

Dissolved Pb data extrapolation

We used dPb data from the entrance of Lancaster Strait (stations CAA1-CAA3 as per Colombo et al. 2019) to calculate dPb fluxes across Lancaster Sound, Nares Strait and Jones Sound. This is because Nares Strait and Jones Sound lack data for dPb. The overall water mass structure in Nares Strait is, however, similar to Lancaster Sound with a clear signal of low temperature Arctic outflow and some contribution from warmer and saltier recirculating Atlantic Water (Supplementary Figure 9). This similarity in water mass structure provides support for using samples collected in Lancaster Sound to provide an estimate of Pb transport across Nares Strait. Jones Sound has a similar structure to Lancaster Sound, yet does not show a strong

signal of recirculating Atlantic Water. The separation of dPb data according to water layers (<47 m, 47-92 m, >92 m) takes account of differences in transport rates of dPb from Arctic Ocean outflow and recirculating Atlantic Water among the gateways. Calculated dPb fluxes are summarized in Supplementary Table 10.

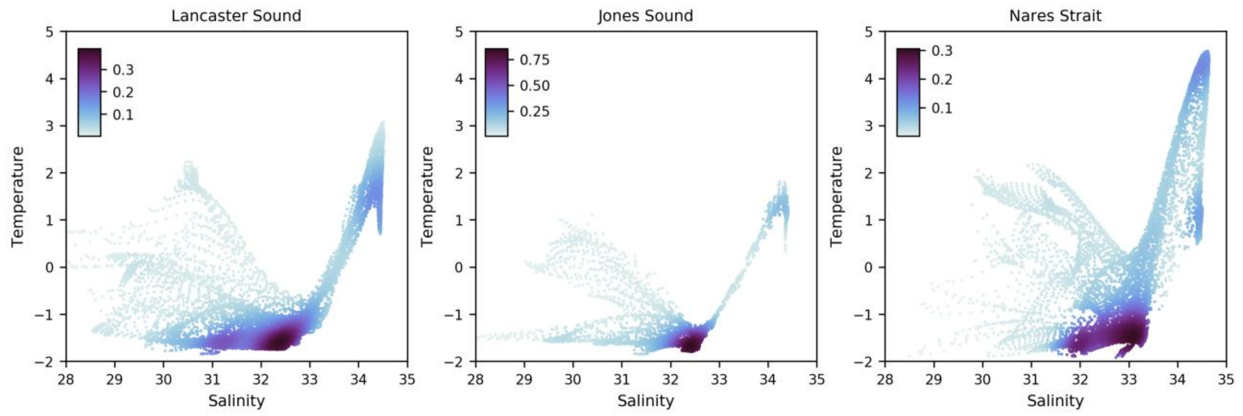

Supplementary Figure 9: Temperature-salinity diagrams of all grid points to full depth along the boundary sections of Lancaster Strait, Nares Strait, and Jones Sound (boundary sections as indicated in Supplementary Figure 8) from monthly averages of September 2015 to August 2016. Colors reflect the number of grid points (as density) that cluster around that temperature and salinity.

Supplementary Table 10: Monthly mean estimates of dPb fluxes (in $Mg \cdot a^{-1}$) across the Canadian Arctic Archipelago through Nares Strait (NS), Lancaster Strait (LS), Jones Sound (JS), and the combined flux of NS, LS and JS. Annual mean net dPb flux (AVG) calculated as average of monthly fluxes; the corresponding uncertainty (STD) is reported as standard deviation (1σ) to monthly variations. Negative (positive) values indicate Arctic export to (import from) the high latitude North Atlantic Ocean. Calculations include conversion of dPb concentration data from $pmol \cdot kg^{-1}$ to $pmol \cdot L^{-1}$ by the density of seawater ($1.023\ kg \cdot L^{-1}$ as per ref.[10]).

Month	NS	LS	JS	Combined
Sep 15	-63	-33	-0.8	-97
Oct 15	-60	-48	0.4	-108
Nov 15	-43	-28	-0.2	-71
Dec 15	-52	-39	-1.7	-93
Jan 16	-56	-30	-0.4	-87
Feb 16	-54	-7	1.3	-60
Mar 16	-69	-23	-2.0	-94
Apr 16	-44	0	1.1	-43
May 16	-54	-19	0.6	-73
Jun 16	-48	-39	-3.2	-90
Jul 16	-42	-47	0.4	-89
Aug 16	-47	-54	-1.3	-103
**AVG**	-53	-31	-0.5	-84
**STD**	8	16	1.3	18

Riverine dPb discharge to the Arctic Ocean

Riverine dPb discharge to the Arctic Ocean was calculated from annual mean volume discharge rates and dissolved Pb concentrations for all rivers where data on both was available until July 2024 (Supplementary Table 11). Volume flux data was obtained from ref.[26] simulating mean riverine discharges for the years 1982-2018 based on remotely-sensed Arctic Discharge Reanalysis (RADR) with years of *in-situ* data. Dissolved Pb data was obtained for the largest Arctic Ocean rivers after thorough literature research on the matter. Our synthesis provides an estimate for ~53% (2715 $km^3 \cdot a^{-1}$) of Arctic Ocean riverine discharge (5169 $km^3 \cdot a^{-1}$) as per ref.[26]. Extrapolated to the total riverine discharge of 5169 $km^3 \cdot a^{-1}$, the Arctic Ocean receives 344 ± 222 Mg dPb per year.

Supplementary Table 11: Compilation of volume flux data (V), dissolved Pb (dPb) concentrations and calculated dPb fluxes (D) for rivers draining into the Arctic Ocean. Errors in dPb fluxes were calculated as the sum of errors of individual contributions (error propagation). [1] Values not included in dPb flux estimate because of limited data coverage and unusually low dPb concentrations. Comparative values only, added for completion. [2] Average of Kuunga, Glacier, Marcil Creek, Saaqu, Devon Island, Cunningham, Garnier, Mecham, Creswell, Le Feuvre Inlet, Pasley, Simpson, Ellice and Tree Rivers, some of which are influenced by local, geogenic sources of dPb including Pb from mining projects.

River	V^A [$km^3 \cdot a^{-1}$]	in-situ data	dPb [$nmol \cdot L^{-1}$]	Range [$nmol \cdot L^{-1}$]	Sampling Date	Pore Size	Comments	Ref.	D [$Mg \cdot a^{-1}$]
**Yenisey**	637 ± 62	1984-2018	0.35 ± 0.28	0.05-0.92	20.08.1998	< 0.4 µm	0.5-1 m depth, n = 8	27	**46.2 ± 37.2**
				0.025-0.029[1]	*09.1993*	*< 0.4 µm*	*dPb range given only, <10 m depth*	28	
**Lena**	577 ± 56	1984-2018	0.35 ± 0.06	0.29-0.40	09.1991	< 0.4 µm	Sal ≤ 0.1, 0.5 m depth, n = 2	29	**63.8 ± 36.8**
			0.72 ± 0.55	0.19-2.22	31.05. – 12.06.1996	< 0.4 µm	Sal ≤ 0.2, <5 m depth, n = 13	30	
				0.070-0.090[1]	*04-14.09.1989*	*< 0.4 µm*	*n = 2*	31	
**Ob**	414 ± 40	1984-2018	0.23 ± 0.12	0.10-0.43	05.08.1998	< 0.4 µm	0.5-1 m depth, n = 7	27	**19.7 ± 10.5**
				0.055-0.083[1]	*09.1993*	*< 0.4 µm*	*dPb range given only, <10 m depth.*	28	
**Mackenzie**	310 ± 30	1984-2017	0.15 ± 0.03	0.123-0.180	21-24.06.2016	< 0.2 µm	Sal ≤ 0.15, 0.5 m depth, n = 6	32	**9.9 ± 2.0**
**Yukon**	212 ± 20	1984-96, 2001-18	0.48 ± 0.39	0.08-1.21	02.04.-24.09.2002, 07.04.-22.09.2004, 17.03.-27.09.2005	< 0.4 µm	Draining into the Bering Sea, n = 31	33–35	**20.9 ± 17.1**
**Pechora**	164 ± 16	1984-90	0.24 ± 0.14	0.05-0.48	17.07.1998	< 0.4 µm	0.5-1 m depth, n = 8	27	**8.2 ± 4.8**
**Severnaya**	106 ± 10	1984-2000, 2007-08, 2010-18	0.35 ± 0.26	0.05-0.77	24.06.1998	< 0.4 µm	0.5-1 m depth, n = 8	27	**10.1 ± 6.3**
			0.57 ± 0.30	0.25-1.26	07.2010 & 07.2012	<0.22 µm	Sal ≤ 0.2, 0.5 m depth, n = 8	36	
**Mezen**	26 ± 2	1984-99, 2017-18	0.21 ± 0.11	0.05-0.39	10.07.1998	< 0.4 µm	0.5-1 m depth, n = 8	27	**1.1 ± 0.6**
**Canadian Arctic Archipelago**	269 ± 26	/	0.014 ± 0.022	0.001-0.073	08.2015	< 0.2 µm	n = 23[2]	37	**0.8 ± 1.2**
**Σ**	**2715 ± 262**								**181 ± 117**

Aerosol Pb deposition into the surface Arctic Ocean

The atmospheric Pb deposition flux into the surface Arctic Ocean was estimated from bulk Pb deposition fluxes calculated for the Eastern, Western, Central and Canadian Arctic (Supplementary Table 12). Our compilation includes the most recent Pb deposition flux estimates for the region obtained during GEOTRACES expeditions GN01 (Western Arctic Ocean), GN02/GN03 (Canadian Arctic) and GN04 (Eastern and Central Arctic Ocean). For our estimate on the entire Arctic Ocean, we use the average of the Pb deposition flux averages presented in Supplementary Table 12 (2.4 ± 1.8 $nmol \cdot m^{-2} \cdot d^{-1}$) multiplied by the surface area of the Arctic Ocean ($9.5 \cdot 10^{12}$ m^2, including the Central Arctic Ocean Basins and the Barents, White, Kara, Laptev, East Siberian, Chukchi, Beaufort and Lincoln Seas) as per Jakobsson (2002)[38]. This yields an estimate of 1729 ± 1296 $Mg \cdot a^{-1}$ of atmospheric Pb deposited into the surface Arctic Ocean.

Supplementary Table 12: Literature values of atmospheric Pb deposition fluxes for the Arctic between 2011-2015.

		**Pb deposition flux [$nmol \cdot m^{-2} \cdot d^{-1}$]**				
**Location**	**Sampling**	**AVG**	**STD**	**Min**	**Max**	**Reference**
Eastern & Central Arctic Ocean	13 Aug - 21 Sep 2011			0.5	6.8	Kadko et al. (2016)[39] [A]
Western Arctic Ocean	9 Aug - 12 Oct 2015	3.4	1.7	1.0	6.6	Kadko et al. (2019)[40] [B]
				0.08	5.96	Marsay et al. (2018)[41] [C]
Canadian Arctic	Jul-Sep 2015	0.3	0.3	0.05	1.3	De Vera et al. (2021)[42] [D]
	Apr-May 2013, Apr-May 2014	4.8	1.5	2.4	7.2	
Alert (Canada)	Jun-Jul 2013, Jun-Jul 2014	1.1	1.0	0.1	2.6	

[A] Pb flux quantified from multiplying the aerosol concentration (averaged over seasonal and annual time scales) by effective deposition velocity derived from a ^{7}Be model.

[B] Pb flux estimated from atmospheric ^{7}Be flux and Pb-^{7}Be ratio of aerosols.

[C] Pb flux estimated from bulk (dry and wet) atmospheric deposition velocity (derived from aerosol ^{7}Be) and the ^{7}Be inventory associated with recent snowfall.

[D] Pb flux quantified from aerosol Pb concentration of the Canadian Arctic and Alert using the average bulk deposition velocity from Kadko et al. (2016)[39] and Marsay et al. (2018)[41].

References

1. Bruland Research Lab. Consensus Values for the GEOTRACES 2008 and SAFe Reference Samples. https://websites.pmc.ucsc.edu/~kbruland/GeotracesSaFe/kwbGeotracesSaFe.html (2009).

2. Rapp, I., Schlosser, C., Rusiecka, D., Gledhill, M. & Achterberg, E. P. Automated preconcentration of Fe, Zn, Cu, Ni, Cd, Pb, Co, and Mn in seawater with analysis using high-resolution sector field inductively-coupled plasma mass spectrometry. *Anal. Chim. Acta* **976**, 1–13 (2017).

3. Wuttig, K. *et al.* Critical evaluation of a seaFAST system for the analysis of trace metals in marine samples. *Talanta* **197**, 653–668 (2019).

4. Galer, S. J. G. & Abouchami, W. Practical application of lead triple spiking for correction of instrumental mass discrimination. in *Goldschmidt Conference Toulouse 1998* 491–492 (1998).

5. Griffiths, A. *et al.* Evaluation of Optimized Procedures for High-Precision Lead Isotope Analyses of Seawater by Multiple Collector Inductively Coupled Plasma Mass Spectrometry. *Anal. Chem.* **92**, 11232–11241 (2020).

6. Tsubouchi, T. *et al.* The Arctic Ocean seasonal cycles of heat and freshwater fluxes: Observation-based inverse estimates. *J. Phys. Oceanogr.* **48**, 2029–2055 (2018).

7. Colombo, M., Rogalla, B., Myers, P. G., Allen, S. E. & Orians, K. J. Tracing dissolved lead sources in the Canadian Arctic: Insights from the Canadian GEOTRACES Program. *ACS Earth Sp. Chem.* **3**, 1302–1314 (2019).

8. Gerringa, L. J. A. *et al.* Dissolved Cd, Co, Cu, Fe, Mn, Ni, Pb and Zn in the Arctic Ocean. *NIOZ Royal Netherlands Institute for Sea Research* (2021) doi:10.25850/nioz/7b.b.jc.

9. Krisch, S. *et al.* Dissolved trace elements (Fe, Mn, Co, Ni, Cu, Zn, Cd and Pb) measured on water bottle samples from ultra clean CTD/Water sampler-system during POLARSTERN cruise PS100/GN05 (ARK-XXX/2). *Alfred Wegener Institute, Helmholtz Centre for Polar and Marine Research, Bremerhaven, PANGAEA* https://doi.pangaea.de/10.1594/PANGAEA.933431 (2021) doi:10.1594/PANGAEA.933431.

10. Woodgate, R. A. Increases in the Pacific inflow to the Arctic from 1990 to 2015, and insights into seasonal trends and driving mechanisms from year-round Bering Strait mooring data. *Prog. Oceanogr.* **160**, 124–154 (2018).

11. Wefing, A.-M., Casacuberta, N., Christl, M., Gruber, N. & Smith, J. N. Circulation timescales of Atlantic Waters in the Arctic Ocean determined from anthropogenic radionuclides. *Ocean Sci.* **17**, 111–129 (2021).

12. Smith, J. N., McLaughlin, F. A., Smethie, W. M. J., Moran, S. B. & Lepore, K. Iodine-129 , 137Cs , and CFC-11 tracer transit time distributions in the Arctic Ocean. *J. Geophys. Res.* **116**, (2011).

13. GEOTRACES Intermediate Data Product Group. The GEOTRACES Intermediate Data Product 2021v2 (IDP2021). at https://doi.org/10.5285/ff46f034-f47c-05f9-e053-6c86abc0dc7e (2021).

14. De Vera, J. *et al.* Anthropogenic lead pervasive in Canadian Arctic seawater. *Proc. Natl. Acad. Sci. U. S. A.* **118**, e2100023118 (2021).

15. Olivelli, A., Krisch, S., Achterberg, E. P., van de Flierdt, T. & Rehkämper, M. Dissolved Pb isotope composition (206Pb/204Pb, 206Pb/207Pb, 207Pb/204Pb, 208Pb/204Pb, 208Pb/207Pb) measured on pooled water samples from POLARSTERN cruise PS100 / GN05 (ARK-XXX/2)

[dataset]. *Alfred Wegener Institute, Helmholtz Centre for Polar and Marine Research, Bremerhaven, PANGAEA* https://doi.pangaea.de/10.1594/PANGAEA.968782 (2024) doi:10.1594/PANGAEA.968782.

16. Rudels, B. *et al.* The interaction between waters from the Arctic Ocean and the Nordic Seas north of Fram Strait and along the East Greenland Current: Results from the Arctic Ocean-02 Oden expedition. *J. Mar. Syst.* **55**, 1–30 (2005).
17. Jensen, L. T. *et al.* Biogeochemical Cycling of Dissolved Zinc in the Western Arctic (Arctic GEOTRACES GN01). *Global Biogeochem. Cycles* **33**, 343–369 (2019).
18. Zurbrick, C. M. *et al.* Dissolved Pb and Pb isotopes in the North Atlantic from the GEOVIDE transect (GEOTRACES GA01) and their decadal evolution. *Biogeosciences* **15**, 4995–5014 (2018).
19. Kelly, A. E., Reuer, M. K., Goodkin, N. F. & Boyle, E. A. Lead concentrations and isotopes in corals and water near Bermuda, 1780-2000. *Earth Planet. Sci. Lett.* **283**, 93–100 (2009).
20. Petrova, M. V *et al.* Mercury species export from the Arctic to the Atlantic Ocean. *Mar. Chem.* **225**, 103855 (2020).
21. Krisch, S. *et al.* Arctic – Atlantic Exchange of the Dissolved Micronutrients Iron, Manganese, Cobalt, Nickel, Copper and Zinc With a Focus on Fram Strait. *Global Biogeochem. Cycles* **36**, e2021GB007191 (2022).
22. Gerringa, L. J. A. *et al.* Dissolved Cd, Co, Cu, Fe, Mn, Ni and Zn in the Arctic Ocean. *J. Geophys. Res. Ocean.* **126**, e2021JC017323 (2021).
23. Colombo, M. *et al.* Canadian Arctic Archipelago Shelf-Ocean Interactions: A Major Iron Source to Pacific Derived Waters Transiting to the Atlantic. *Global Biogeochem. Cycles* **35**, e2021GB007058 (2021).
24. Zhang, Y. *et al.* Studies of the Canadian Arctic Archipelago water transport and its relationship to basin-local forcings: Results from AO-FVCOM. *J. Geophys. Res. Ocean.* **121**, 4392–4415 (2016).
25. Grivault, N., Hu, X. & Myers, P. G. Impact of the Surface Stress on the Volume and Freshwater Transport Through the Canadian Arctic Archipelago From a High-Resolution Numerical Simulation. *J. Geophys. Res. Ocean.* **123**, 9038–9060 (2018).
26. Feng, D. *et al.* Recent changes to Arctic river discharge. *Nat. Commun.* **12**, 6917 (2021).
27. Guay, C. K. H. *et al.* Measurements of Cd, Cu, Pb and Zn in the lower reaches of major Eurasian arctic rivers using trace metal clean techniques. *Environ. Pollut.* **158**, 624–630 (2010).
28. Dai, M. H. & Martin, J. M. First data on trace metal level and behaviour in two major Arctic river-estuarine systems (Ob and Yenisey) and in the adjacent Kara Sea, Russia. *Earth Planet. Sci. Lett.* **131**, 127–141 (1995).
29. Guieu, C., Huang, W. W., Martin, J. M. & Yong, Y. Y. Outflow of trace metals into the Laptev Sea by the Lena River. *Mar. Chem.* **53**, 255–267 (1996).
30. Hölemann, J. A., Schirmacher, M. & Prange, A. Seasonal variability of trace metals in the Lena River and the southeastern Laptev Sea: Impact of the spring freshet. *Glob. Planet. Change* **48**, 112–125 (2005).
31. Martin, J. M., Guan, D. M., Elbaz-Poulichet, F., Thomas, A. J. & Gordeev, V. V. Preliminary assessment of the distributions of some trace elements (As, Cd, Cu, Fe, Ni, Pb and Zn) in a pristine aquatic environment: The Lena River estuary (Russia). *Mar. Chem.* **43**, 185–199 (1993).
32. Kipp, L. E., Henderson, P. B., Wang, Z. A. & Charette, M. A. Deltaic and Estuarine Controls on

Mackenzie River Solute Fluxes to the Arctic Ocean. *Estuaries and Coasts* **43**, 1992–2014 (2020).

33. Schuster, P. F. *Water and Sediment Quality in the Yukon River Basin, Alaska, During Water Year 2002*. *Open-File Report 2005-1199* http://pubsdata.usgs.gov/pubs/of/2005/1199/ (2005).

34. Schuster, P. F. *Water and Sediment Quality in the Yukon River Basin, Alaska, During Water Year 2004*. *Open-File Report 2006-1258* https://pubs.usgs.gov/publication/ofr20061258 (2006) doi:10.3133/ofr20061258.

35. Schuster, P. F. *Water and Sediment Quality in the Yukon River Basin, Alaska, During Water Year 2005*. *Open-File Report 2007-1037* https://pubs.usgs.gov/publication/ofr20071037 (2007) doi:10.3133/ofr20071037.

36. Pokrovsky, O. S. *et al.* Fate of colloids during estuarine mixing in the Arctic. *Ocean Sci.* **10**, 107–125 (2014).

37. Colombo, M., Brown, K. A., Vera, J. De, Bergquist, B. A. & Orians, K. J. Trace metal geochemistry of remote rivers in the Canadian Arctic Archipelago. *Chem. Geol.* **525**, 479–491 (2019).

38. Jakobsson, M. Hypsometry and volume of the Arctic Ocean and its constituent seas. *Geochemistry, Geophys. Geosystems* **3**, (2002).

39. Kadko, D., Galfond, B., Landing, W. M. & Shelley, R. U. Determining the pathways, fate, and flux of atmospherically derived trace elements in the arctic ocean/ice system. *Mar. Chem.* **182**, 38–50 (2016).

40. Kadko, D. *et al.* The residence times of trace elements determined in the surface Arctic Ocean during the 2015 US Arctic GEOTRACES expedition. *Mar. Chem.* **208**, 56–69 (2019).

41. Marsay, C. M. *et al.* Concentrations, provenance and flux of aerosol trace elements during US GEOTRACES Western Arctic cruise GN01. *Chem. Geol.* **502**, 1–14 (2018).

42. De Vera, J. *et al.* Amount, Sources, and Dissolution of Aerosol Trace Elements in the Canadian Arctic. *ACS Earth Sp. Chem.* **5**, 2686–2699 (2021).

Plotting

Supplementary Figures 1-4 were made by SK using Ocean Data View, version 5.7.2 (Schlitzer, R., Ocean Data View, https://odv.awi.de, 2024). Supplementary Figures 5 and 6 were made by SK with Microsoft Excel (version 2019, https://office.microsoft.com/excel). Supplementary Figure 7 was produced by SK with Origin Pro software (Version 2024. OriginLab Corporation, Northampton, MA, USA). Supplementary Figures 8 and 9 were made by BR with Python software, version 3.7 (Python Software Foundation, https://www.python.org/).
