# Supplementary material for: The Arctic Ocean is a net sink for anthropogenic lead deposited into the Atlantic Ocean
Source: Nat Commun. 2025 Dec 17;16:11238. doi: 10.1038/s41467-025-67620-9 (PMC12714867; doi:10.1038/s41467-025-67620-9)
Supplement: Supplementary file 3 — Reporting Summary [file 41467_2025_67620_MOESM3_ESM.pdf]

nature portfolio

Corresponding author(s): Stephan Krisch (stephan.krisch@tu-braunschweig.de)

Last updated by author(s): Oct 30, 2025

Reporting Summary

Nature Portfolio wishes to improve the reproducibility of the work that we publish. This form provides structure for consistency and transparency in reporting. For further information on Nature Portfolio policies, see our Editorial Policies and the Editorial Policy Checklist.

Statistics

For all statistical analyses, confirm that the following items are present in the figure legend, table legend, main text, or Methods section.

n/a	Confirmed	
☐	☒	The exact sample size (n) for each experimental group/condition, given as a discrete number and unit of measurement
☐	☒	A statement on whether measurements were taken from distinct samples or whether the same sample was measured repeatedly
☐	☒	The statistical test(s) used AND whether they are one- or two-sided<br>*Only common tests should be described solely by name; describe more complex techniques in the Methods section.*
☒	☐	A description of all covariates tested
☒	☐	A description of any assumptions or corrections, such as tests of normality and adjustment for multiple comparisons
☐	☒	A full description of the statistical parameters including central tendency (e.g. means) or other basic estimates (e.g. regression coefficient) AND variation (e.g. standard deviation) or associated estimates of uncertainty (e.g. confidence intervals)
☐	☒	For null hypothesis testing, the test statistic (e.g. F, t, r) with confidence intervals, effect sizes, degrees of freedom and P value noted<br>*Give P values as exact values whenever suitable.*
☒	☐	For Bayesian analysis, information on the choice of priors and Markov chain Monte Carlo settings
☒	☐	For hierarchical and complex designs, identification of the appropriate level for tests and full reporting of outcomes
☐	☒	Estimates of effect sizes (e.g. Cohen's d, Pearson's r), indicating how they were calculated

Our web collection on statistics for biologists contains articles on many of the points above.

Software and code

Policy information about availability of computer code

Data collection	Volume transport across Nares Strait, Lancaster Sound, and Jones Sound (all Canadian Arctic, as visualized in Supplementary Figure 8) were obtained by using 5-day average dynamics fields from the reference experiment of the ANHA12 configuration within the Nucleus for European Modeling of the Ocean (NEMO, https://canadian-nemo-ocean-modelling-forum-commuity-of-practice.readthedocs.io/en/latest/Institutions/UofA/Configurations/ANHA12/index.html).<br>All other volume fluxes were published and obtained by Tsubouchi et al (2018, 10.1175/JPO-D-17-0239.1).
Data analysis	Oceanographic data was analysed with Ocean Data View 2024 software and DIVA gridding calculations (version 5.7.2). Correlation analyses are conducted with Origin Pro software (Version 2024. OriginLab Corporation, Northampton, MA, USA) and Microsoft Excel.

For manuscripts utilizing custom algorithms or software that are central to the research but not yet described in published literature, software must be made available to editors and reviewers. We strongly encourage code deposition in a community repository (e.g. GitHub). See the Nature Portfolio guidelines for submitting code & software for further information.

Data

Policy information about availability of data

All manuscripts must include a data availability statement. This statement should provide the following information, where applicable:

- Accession codes, unique identifiers, or web links for publicly available datasets
- A description of any restrictions on data availability
- For clinical datasets or third party data, please ensure that the statement adheres to our policy

All data used throughout this publication is accessible online.
Physical oceanography data can be obtained from:
https://doi.pangaea.de/10.1594/PANGAEA.871025 (PS100/GN05 large CTD, ref. 93),
https://doi.pangaea.de/10.1594/PANGAEA.871030 (PS100/GN05 clean CTD, ref. 94),
https://doi.pangaea.de/10.1594/PANGAEA.859558 (PS94/GN04 large and clean CTD, ref. 95).
Canadian Arctic Archipelago volume fluxes are calculated from a simulation with the ANHA12 configuration of the NEMO ocean model, which can be obtained from:
https://canadian-nemo-ocean-modelling-forum-commuity-of-practice.readthedocs.io/en/latest/Institutions/UofA/Configurations/ANHA12/index.html
Pb data can be obtained from:
https://doi.pangaea.de/10.1594/PANGAEA.933431 (dissolved Pb from GN05, ref. 96),
https://doi.pangaea.de/10.1594/PANGAEA.968782 (Pb isotope composition from GN05, ref. 97),
https://doi.org/10.25850/nioz/7b.b.jc (dissolved Pb from GN04, ref. 98).

Research involving human participants, their data, or biological material

Policy information about studies with human participants or human data. See also policy information about sex, gender (identity/presentation), and sexual orientation and race, ethnicity and racism.

Reporting on sex and gender	N/A
Reporting on race, ethnicity, or other socially relevant groupings	N/A
Population characteristics	N/A
Recruitment	N/A
Ethics oversight	N/A

Note that full information on the approval of the study protocol must also be provided in the manuscript.

Field-specific reporting

Please select the one below that is the best fit for your research. If you are not sure, read the appropriate sections before making your selection.

☐ Life sciences ☐ Behavioural & social sciences ☒ Ecological, evolutionary & environmental sciences

For a reference copy of the document with all sections, see nature.com/documents/nr-reporting-summary-flat.pdf

Life sciences study design

All studies must disclose on these points even when the disclosure is negative.

Sample size	*Describe how sample size was determined, detailing any statistical methods used to predetermine sample size OR if no sample-size calculation was performed, describe how sample sizes were chosen and provide a rationale for why these sample sizes are sufficient.*
Data exclusions	*Describe any data exclusions. If no data were excluded from the analyses, state so OR if data were excluded, describe the exclusions and the rationale behind them, indicating whether exclusion criteria were pre-established.*
Replication	*Describe the measures taken to verify the reproducibility of the experimental findings. If all attempts at replication were successful, confirm this OR if there are any findings that were not replicated or cannot be reproduced, note this and describe why.*
Randomization	*Describe how samples/organisms/participants were allocated into experimental groups. If allocation was not random, describe how covariates were controlled OR if this is not relevant to your study, explain why.*
Blinding	*Describe whether the investigators were blinded to group allocation during data collection and/or analysis. If blinding was not possible, describe why OR explain why blinding was not relevant to your study.*

Behavioural & social sciences study design

All studies must disclose on these points even when the disclosure is negative.

Study description	*Briefly describe the study type including whether data are quantitative, qualitative, or mixed-methods (e.g. qualitative cross-sectional, quantitative experimental, mixed-methods case study).*
Research sample	*State the research sample (e.g. Harvard university undergraduates, villagers in rural India) and provide relevant demographic information (e.g. age, sex) and indicate whether the sample is representative. Provide a rationale for the study sample chosen. For studies involving existing datasets, please describe the dataset and source.*
Sampling strategy	*Describe the sampling procedure (e.g. random, snowball, stratified, convenience). Describe the statistical methods that were used to predetermine sample size OR if no sample-size calculation was performed, describe how sample sizes were chosen and provide a rationale for why these sample sizes are sufficient. For qualitative data, please indicate whether data saturation was considered, and what criteria were used to decide that no further sampling was needed.*
Data collection	*Provide details about the data collection procedure, including the instruments or devices used to record the data (e.g. pen and paper, computer, eye tracker, video or audio equipment) whether anyone was present besides the participant(s) and the researcher, and whether the researcher was blind to experimental condition and/or the study hypothesis during data collection.*
Timing	*Indicate the start and stop dates of data collection. If there is a gap between collection periods, state the dates for each sample cohort.*
Data exclusions	*If no data were excluded from the analyses, state so OR if data were excluded, provide the exact number of exclusions and the rationale behind them, indicating whether exclusion criteria were pre-established.*
Non-participation	*State how many participants dropped out/declined participation and the reason(s) given OR provide response rate OR state that no participants dropped out/declined participation.*
Randomization	*If participants were not allocated into experimental groups, state so OR describe how participants were allocated to groups, and if allocation was not random, describe how covariates were controlled.*

Ecological, evolutionary & environmental sciences study design

All studies must disclose on these points even when the disclosure is negative.

Study description	We use dissolved lead (dPb) water column data to calculate dPb fluxes between the Arctic Ocean and the Atlantic Ocean. Anthropogenic Pb emissions were traced through the use of dPb isotope composition (206Pb/207Pb vs. 208Pb/207Pb).
Research sample	We collected dissolved (<0.2 µm) lead samples from discrete water depths across Fram Strait, the Barents Sea Opening, and the Canadian Arctic. The locations of stations and sampling depths were chosen according to water mass properties and aimed to capture the prevailing water masses in high-resolution with up to 24 depths per station from surface to bottom.
Sampling strategy	Dissolved Pb (dPb) and dPb isotope composition samples were collected and preserved under trace metal clean conditions following GEOTRACES guidelines. Conductivity, temperature, and pressure were recorded from the rosette-CTD unit. The CTD data was used to indentify water masses.
Data collection	All samples were analysed, and data was collected, strictly following GEOTRACES guidelines. All samples were analysed under trace-element clean conditions after pre-concentrations and subsequent high-resolution inductively coupled plasma mass spectrometry (HR-ICP-MS).<br>Fram Strait dissolved Pb (dPb) samples were shipped and analysed by Stephan Krisch at GEOMAR in Kiel (Germany). Fram Strait dPb isotope samples were measured at the Imperial College London (UK) by Arianna Olivelli. Dissolved Pb samples from the Barents Sea Opening were shipped and analysed by Loes Gerringa and Rob Middag at NIOZ in Texel (The Netherlands). Canadian Arctic dPb samples were shipped and analysed at the University of British Columbia (Canada). The Canadian Arctic dataset is discussed and has been published by Colombo et al. (2019, 10.1021/acsearthspacechem.9b00083). Canadian Arctic Pb isotope samples were analysed by - and is discussed in - De Vera et al. (2021, 10.1073/pnas.2100023118).
Timing and spatial scale	Fram Strait was sampled between 21 July–6 September 2016. The Barents Sea Opening was sampled between 17 August–15 October 2015. The Canadian Arctic was sampled between 10 July-1 October 2015.<br>The location of stations (Latitude/Longitude) and sample depths are published with the corresponding datasets.
Data exclusions	Data with quality flags 3 ('probably bad value'), 4 ('bad value'), 6 ('value below detection'), and 9 ('missing value') according to the SeaDataNet Scheme were excluded.
Reproducibility	Accuracy and precision during the analyses were conducted through the use of reference materials, and through inter-lab comparsion of triplicate samples ('intercalibration') following GEOTRACES guidelines. Please see individual data reports for details.
Randomization	Samples were run batch-wise, typically from (expected) low to high concentrations to prevent cross-contamination ('carry-over').

Blinding	Blinding not relevant as the study region was not sampled (and analysed) for Pb previously.

Did the study involve field work? ☒ Yes ☐ No

Field work, collection and transport

Field conditions	Fieldwork at sea was conducted in Arctic summer. The sampling was independent from weather conditions.
Location	The study region encompasses the Fram Strait, Barents Sea, and Canadian Arctic Archipelago.<br>The location of stations (Latitude/Longitude) and sample depths are published in the corresponding datasets.
Access & import/export	Samples were collected strictly following international laws on environmental protection. This includes export of sample material. Permits to sample the Canadian, Greenlandic, and Norwegian (incl. Svalbard) EEZs were obtained and are hold by the expedition leaders (see corresponding expedition reports).
Disturbance	Disturbance to the study region was minimized by limiting noise, CO2 emissions, and through the re-use of materials (also sample material) where possible. The compliance to health and safety and environmental protection laws was frequently assessed.

Reporting for specific materials, systems and methods

We require information from authors about some types of materials, experimental systems and methods used in many studies. Here, indicate whether each material, system or method listed is relevant to your study. If you are not sure if a list item applies to your research, read the appropriate section before selecting a response.

Materials & experimental systems

n/a	Involved in the study	
☐	☐	Antibodies
☐	☐	Eukaryotic cell lines
☐	☐	Palaeontology and archaeology
☐	☐	Animals and other organisms
☐	☐	Clinical data
☐	☐	Dual use research of concern
☐	☐	Plants

Methods

n/a	Involved in the study	
☐	☐	ChIP-seq
☐	☐	Flow cytometry
☐	☐	MRI-based neuroimaging

Antibodies

Antibodies used	N/A
Validation	N/A

Eukaryotic cell lines

Policy information about cell lines and Sex and Gender in Research

Cell line source(s)	N/A
Authentication	N/A
Mycoplasma contamination	N/A
Commonly misidentified lines<br>(See ICLAC register)	N/A

Palaeontology and Archaeology

Specimen provenance	N/A
Specimen deposition	N/A

Dating methods | N/A

☐ Tick this box to confirm that the raw and calibrated dates are available in the paper or in Supplementary Information.

Ethics oversight | N/A

Note that full information on the approval of the study protocol must also be provided in the manuscript.

Animals and other research organisms

Policy information about studies involving animals; ARRIVE guidelines recommended for reporting animal research, and Sex and Gender in Research

Laboratory animals	N/A
Wild animals	N/A
Reporting on sex	N/A
Field-collected samples	N/A
Ethics oversight	N/A

Note that full information on the approval of the study protocol must also be provided in the manuscript.

Clinical data

Policy information about clinical studies

All manuscripts should comply with the ICMJE guidelines for publication of clinical research and a completed CONSORT checklist must be included with all submissions.

Clinical trial registration	N/A
Study protocol	N/A
Data collection	N/A
Outcomes	N/A

Dual use research of concern

Policy information about dual use research of concern

Hazards

Could the accidental, deliberate or reckless misuse of agents or technologies generated in the work, or the application of information presented in the manuscript, pose a threat to:

No	Yes	
☒	☐	Public health
☒	☐	National security
☒	☐	Crops and/or livestock
☒	☐	Ecosystems
☒	☐	Any other significant area

Experiments of concern

Does the work involve any of these experiments of concern:

No	Yes	
☒	☐	Demonstrate how to render a vaccine ineffective
☒	☐	Confer resistance to therapeutically useful antibiotics or antiviral agents
☒	☐	Enhance the virulence of a pathogen or render a nonpathogen virulent
☒	☐	Increase transmissibility of a pathogen
☒	☐	Alter the host range of a pathogen
☒	☐	Enable evasion of diagnostic/detection modalities
☒	☐	Enable the weaponization of a biological agent or toxin
☒	☐	Any other potentially harmful combination of experiments and agents

Plants

Seed stocks	N/A
Novel plant genotypes	N/A
Authentication	N/A

ChIP-seq

Data deposition

☐ Confirm that both raw and final processed data have been deposited in a public database such as GEO.

☐ Confirm that you have deposited or provided access to graph files (e.g. BED files) for the called peaks.

Data access links<br>*May remain private before publication.*	N/A
Files in database submission	N/A
Genome browser session<br>(e.g. UCSC)	N/A

Methodology

Replicates	N/A
Sequencing depth	N/A
Antibodies	N/A
Peak calling parameters	N/A
Data quality	N/A
Software	N/A

Flow Cytometry

Plots

Confirm that:

- [ ] The axis labels state the marker and fluorochrome used (e.g. CD4-FITC).
- [ ] The axis scales are clearly visible. Include numbers along axes only for bottom left plot of group (a 'group' is an analysis of identical markers).
- [ ] All plots are contour plots with outliers or pseudocolor plots.
- [ ] A numerical value for number of cells or percentage (with statistics) is provided.

Methodology

Sample preparation	N/A
Instrument	N/A
Software	N/A
Cell population abundance	N/A
Gating strategy	N/A

- [ ] Tick this box to confirm that a figure exemplifying the gating strategy is provided in the Supplementary Information.

Magnetic resonance imaging

Experimental design

Design type	N/A
Design specifications	N/A
Behavioral performance measures	N/A

Acquisition

Imaging type(s)	N/A
Field strength	N/A
Sequence & imaging parameters	N/A
Area of acquisition	N/A

Diffusion MRI ☐ Used ☒ Not used

Preprocessing

Preprocessing software	N/A
Normalization	N/A
Normalization template	N/A
Noise and artifact removal	N/A
Volume censoring	N/A

Statistical modeling & inference

Model type and settings	N/A
Effect(s) tested	N/A

Specify type of analysis: ☐ Whole brain ☐ ROI-based ☐ Both

Statistic type for inference

(See Eklund et al. 2016)

N/A

Correction

N/A

Models & analysis

n/a	Involved in the study
☒	☐ Functional and/or effective connectivity
☒	☐ Graph analysis
☒	☐ Multivariate modeling or predictive analysis

Functional and/or effective connectivity

Report the measures of dependence used and the model details (e.g. Pearson correlation, partial correlation, mutual information).

Graph analysis

Report the dependent variable and connectivity measure, specifying weighted graph or binarized graph, subject- or group-level, and the global and/or node summaries used (e.g. clustering coefficient, efficiency, etc.).

Multivariate modeling and predictive analysis

Specify independent variables, features extraction and dimension reduction, model, training and evaluation metrics.
